# Evaluating the analgesic effect and advantage of transcutaneous electrical acupoint stimulation combined with opioid drugs for moderate to severe cancer-related pain: a study protocol for a randomized controlled trial

**DOI:** 10.1186/s13063-018-3145-y

**Published:** 2019-01-11

**Authors:** Yi Liang, Guanai Bao, Liyan Gong, Jie Zhou, Xiangming Kong, Ran Ran, Xiaomei Shao, Yongliang Jiang, Weiping Zhang, Boyi Liu, Junying Du, Junfan Fang, Na Nie, Conghua Ji, Jianqiao Fang

**Affiliations:** 1 Department of Acupuncture, The Third Affiliated Hospital of Zhejiang Chinese Medical University, No. 219 Moganshan Road, XiHu District, Hangzhou, 310005 Zhejiang Province China; 2 The Third Clinical Medical College of Zhejiang Chinese Medical University, No. 548 Binwen Road, Binjiang District, Hangzhou, 310053 Zhejiang Province China; 3 The Zhejiang Cancer Hospital, No. 1 Banshan East Road, Gongshu District, Hangzhou, 310022 Zhejiang Province China; 4 The Clinical Research Institute of Zhejiang Provincial Hospital of TCM, No.54 Youdian Road, Xihu District, Hangzhou, 310006 Zhejiang Province China

**Keywords:** Study protocol, Randomized controlled trial, TEAS, Cancer-related pain

## Abstract

**Background:**

Transcutaneous electrical acupoint stimulation (TEAS), which is also known as acupuncture-like transcutaneous electrical nerve stimulation (TENS), has been widely used in acute or chronic pain. However, previous research has not demonstrated that TEAS is effective for cancer-related pain. Opioid drugs are strongly recommended for treating cancer-related pain, but opioid-induced immunosuppression is still the most intractable drug-induced medical problem. Evaluating the efficacy and potential advantage of TEAS combined with opioid drugs in moderate and severe cancer-related pain in China is important because such studies are lacking.

**Methods/Design:**

This trial is a multicenter, prospective randomized controlled clinical trial. In total, 160 patients who were enrolled from two hospitals in the Zhejiang Province (China) will be randomly allocated into two groups: a TEAS group and sham TEAS group without acupoint electrical stimulation. Both groups will receive a 21-day interval of chemotherapy and conventional cancer pain therapy. Fifteen treatment sessions will be performed over a three-week period. The primary outcomes will be measured by changes in the Numerical Rating Scale (NRS) scores and equivalent dosage of morphine at baseline, three weeks of treatment and one two-week follow-up. The secondary outcome measures include cellular immunity function, life quality assessment, opioids side effects assessment, and safety and compliance evaluation.

**Discussion:**

This trial is expected to clarify whether TEAS is effective for cancer-related pain. These results demonstrate the advantage of TEAS combined with opioid drugs on improving immune function and decreasing opioid induced side effects.

**Trial registration:**

Chinese Clinical Trial Registry, ChiCTR-13003803. Registered on 27 August 2013.

**Electronic supplementary material:**

The online version of this article (10.1186/s13063-018-3145-y) contains supplementary material, which is available to authorized users.

## Background

Cancer-related pain, which always manifests as severe and intractable pain, is the most disruptive cancer-related event to the cancer patient’s quality of life [1, 2]. The overall prevalence of pain in patients with cancer had been reported as > 50% [2]. In a 2014 systematic review of 19 studies, the pooled prevalence of breakthrough cancer-related pain was 59.2%, which ranged from 39.9% in outpatient clinics to 80.5% in hospices [3]. A Cochrane systematic review published in 2015 reported that 40% of patients with early or intermediate stage cancer and 90% with advanced cancer suffer from moderate or severe pain [4]. Although the World Health Organization (WHO) has given more attention to control cancer pain for several decades, under-treatment of cancer pain has been widely documented [5]. To date, opiate drugs remain the gold standard for treating moderate to severe pain or breakthrough pain resulting from cancer and are recommended strongly for treating cancer pain [6]. Nevertheless, long-term use of opioid drugs extensively inhibits the immune system [7] by leading to T lymphocyte apoptosis [8], inhibiting activation of T lymphocyte proliferation and secretion of IL-2 [9, 10]. Opioid-induced immunosuppression has become the most significant drug-induced medical problem or side effect of opiate drug administration [11]. In addition, chronic pain (including cancer pain) also has an inhibitory effect on the immune system [12]. At present, an overwhelming majority of cancer patients are simultaneously bothered by both cancer-related pain and opioid-induced immunosuppression.

Acupuncture has been accepted worldwide given its effectiveness in treating various pains. A systematic review published recently demonstrated that acupuncture is effective in relieving cancer-related pain, particularly malignancy-related and surgery-induced pain [13]. Acupuncture plus drug therapy is more effective than conventional drug therapy alone for cancer-related pain [14]. Transcutaneous electrical nerve stimulation (TENS) has been widely used in acute or chronic pain, is effective in 67% of different types of pain [15], and may be a novel treatment for cancer bone pain [16]. However, the analgesic effects of TENS for chronic pain or cancer pain are obscure due to a lack of suitable randomized controlled trials (RCTs) [17, 18]. Transcutaneous electrical acupoint stimulation (TEAS), also called acupuncture-like TENS or acupuncture-type TENS, is a novel therapy combined acupoint stimulation and TENS technique. TEAS has become more popular than acupuncture worldwide given its non-invasive feature. Moreover, it has been reported that the analgesic effect of TEAS was similar to acupuncture on postoperative surgical pain relief in gynecologic oncology patients [19]. Previous clinical studies also demonstrated a positive effect of TEAS analgesia in patients with labor pain and postoperative pain [20–23]. However, there is no direct evidence on the effect of TEAS on cancer-related pain. Moreover, acupuncture and TEAS has immunomodulatory effects [24, 25], but whether TEAS combined with opioid drugs will helpful to improve immune function and reduce other opioid-related side effects remains unclear. Rigorously designed and large multicenter RCTs are required to assess the value and potential advantage of TEAS in the management of cancer-related pain.

## Methods

### Study design

**Fig. 1:**
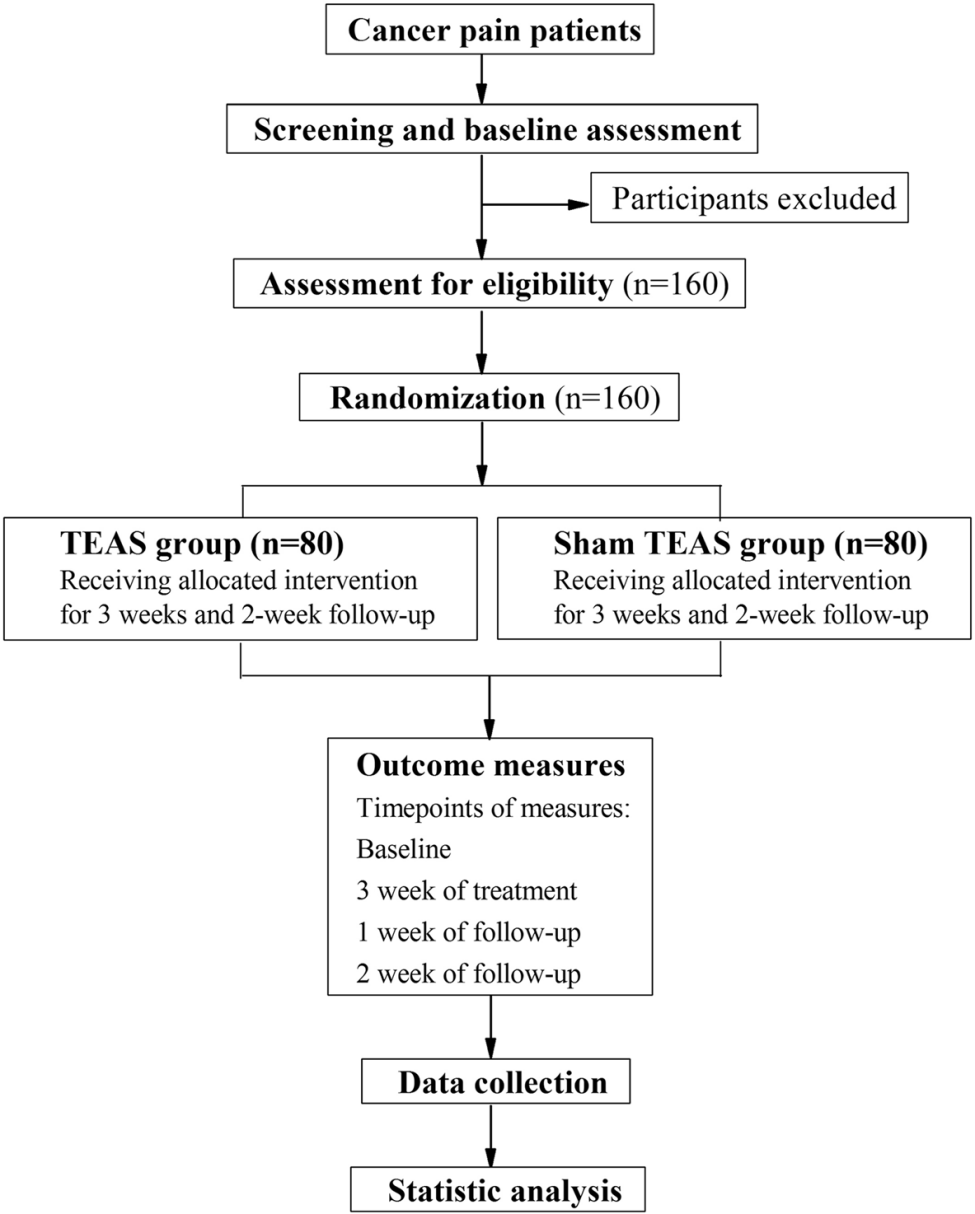
*Flow diagram* of the study design

This multicenter, randomized controlled clinical trial will be performed in two inpatient units and comprises two parallel groups. After the inclusion and consent of the Institutional Review Board, participants will be randomized to either the control (sham TEAS) or TEAS group. The TEAS group will receive conventional cancer therapy (including 21-day interval chemotherapy and three steps analgesic therapy) and 60 additional minutes of TEAS treatment (30 min in each) five days per week for three weeks (15 sessions in total) during the inpatient stay. The control group will receive conventional cancer therapy and sham TEAS (lead wires of the apparatus will be cut down). The trial design is summarized in Fig. 1. The Standard Protocol Items: Recommendations for Interventional Trials (SPIRIT) checklist is provided as Additional file 1.

In all groups, participants will be permitted to use necessary analgesics such as short-acting morphine tablets or morphine injection during breakthrough cancer pain. The type, dose, and time of administration of the agent must be recorded in a cancer pain diary. The trial was registered in ClinicalTrials.gov with approval number ChiCTR-TRC-13003803.

### Participant recruitment

A total of 160 participants will be recruited in the Zhejiang Cancer Hospital and the Third Affiliated Hospital of Zhejiang Chinese Medicine University in Zhejiang Province, China. Our study will be advertised on the Internet and on posters in communities and hospitals. Prospective participants will be informed of the benefit and possible risk associated with this study. Participants will be told that they can withdraw from the trial at any time without specifying reasons. Participants will voluntarily provide written informed consent before enrollment. If prospective participants are interested in participating, they will be invited for a series of assessments by an oncologist. Eligible participants will be randomized into two groups with different treatments once informed consent has been obtained.

### Inclusion criteria

Eligible participants should match the diagnostic criteria for cancer established by the Union for International Cancer Control (UICC) accompanied with pain induced by primary cancer, such as lung cancer, esophageal cancer, mammary cancer, gastric cancer, and pancreatic cancer. Patients must also meet the following criteria: (1) men or women aged 18–70 years with a > 2-month expectant lifespan; (2) a numerical rating scale (NRS) score ≥ 4 or treated by opioid drug therapy; (3) a Karnofsy performance state scale (KPS) score ≥ 60; (4) receiving 21-day intervals of chemotherapy; (5) patients have the ability to estimate themselves (pain, quality of life, etc.); and (6) written informed consent was provided by themselves or their lineal kin.

### Exclusion criteria

Patients receiving other analgesic therapy, such as radiation, bone cement, and nerve block therapy, with pain unrelated to cancer, will not be included. Patients who suffer from active cerebrovascular disease, respiratory depression, severe cognitive impairment, or mental disorders will not be included. Patients who are allergic to opioid drugs and patients with skin lesions in local acupoints will also be excluded.

### Ethical considerations

The protocol of this study was approved by the ethics committees of the Third Affiliated Hospital of Zhejiang Chinese Medical University (permission number: ZSLL-KY-2013-016) and Zhejiang Cancer Hospital (permission number: zjzlyy [2014]-09-86号). The purpose, nature, and potential risks of this study were fully explained to the participants and their families. All participants have provided written informed consent for inclusion in this study.

### Randomization and blinding

To guarantee allocation concealment, randomization will be performed by independent research staff. The random number generated by the SPSS statistical package program (version 20.0, SPSS, Inc., Chicago, IL, USA), and the treatment codes will be placed in sealed opaque envelopes. The study coordinator who does not participate in treatment or nursing was responsible for allocating the randomization codes. In this study, the outcome assessors, doctors-in-charge, data analysts, and participants will be blinded to the group assignments. However, it will be impossible to blind the TEAS therapist because they must be trained to perform the TEAS according to the research plan.

### Interventions

The study is a randomized clinical trial that will be performed in the inpatient oncology ward of two hospitals. Participants will be randomized to either the control or the TEAS group. Both groups will receive 21-days interval chemotherapy according to the NCCN Clinical Practice Guidelines in Oncology (Version 2.2011, details in Table 1) and conventional cancer pain therapy under the guidance of the WHO three-step analgesic ladder principle. The TEAS group will receive 15 additional TEAS sessions, whereas the control group will receive sham stimulation of TEAS during the trial period.

**Table 1:** Details of the 21-day interval chemotherapy regimen

Chemotherapy regimen	Drugs	Dosage	Administration route	Medication time
Combined chemotherapy				
NP	Navelbine	25 mg/m^2^	i.v.	D1, D8
	Cis-platinum	80 mg/m^2^	i.v.	D1
TP	Paclitaxel	135~175 mg/m^2^	i.v.	D1
	Cis-platinum	75 mg/m^2^	i.v.	D1
	or Carboplatin	AUC = 5–6 mg/m^2^	i.v.	D1
GP	Gemcitabine	1250 mg/m^2^	i.v.	D1, D8
	Cis-platinum	75 mg/m^2^	i.v.	D1
	or Carboplatin	AUC = 5–6 mg/m^2^	i.v.	D1
DP	Docetaxel	75 mg/m^2^	i.v.	D1
	Cis-platinum	75 mg/m^2^	i.v.	D1
	or Carboplatin	AUC = 5–6 mg/m^2^	i.v.	D1
PC	Pemetrexed	500 mg/m^2^	i.v.	D1
	Cis-platinum	75 mg/m^2^	i.v.	D1
	or Carboplatin	AUC = 5–6 mg/m^2^	i.v.	D1
EP	Etoposide	100 mg/m^2^	i.v.	D1–3
	Cis-platinum	80 mg/m^2^	i.v.	D1
	or Carboplatin	AUC = 5–6 mg/m^2^	i.v.	D1
EP	Etoposide	120 mg/m^2^	i.v.	D1–3
	Cis-platinum	60 mg/m^2^	i.v.	D1
IP	Irinotecan	65 mg/m^2^	i.v.	D1, D8
	Cis-platinum	30 mg/m^2^	i.v.	D1, D8
AP	Amrubicin	40 mg/m^2^	i.v.	D1–3
	Cis-platinum	60 mg/m^2^	i.v.	D1
XT	Docetaxel	75 mg/m^2^	i.v.	D1
	Capecitabine	950 mg/m^2^	p.o.	D1–14
GT	Paclitaxel	175 mg/m^2^	i.v.	D1
	Gemcitabine	1000~1250 mg/m^2^	i.v.	D1, D8
TC	Docetaxel	75 mg/m^2^	i.v.	D1
	Cyclophosphamide	600 mg/m^2^	i.v.	D1
TAC	Docetaxel	75 mg/m^2^	i.v.	D1
	Doxorubicin	50 mg/m^2^	i.v.	D1
	Cyclophosphamide	500 mg/m^2^	i.v.	D1
Single-agent chemotherapy				
Doxorubicin	Doxorubicin	75 mg/m^2^	i.v.	D1
Paclitaxel	Paclitaxel	175 mg/m^2^	i.v.	D1
Docetaxel	Docetaxel	80 mg/m^2^	i.v.	D1
Capecitabine	Capecitabine	1000–1250 mg/m^2^	p.o.	D1–14
Herceptin	Herceptin	6 mg/kg	i.v.	D21

*i.v.* intravenous injection, *p.o.* per os (oral administration)

#### TEAS group

The TEAS treatment strategy is designed by an experienced acupuncturist with > 30 years of practice experience in acupuncture analgesia. TEAS will be performed by two doctors from the acupuncture ward who have a master’s degree. All oncologists who enroll participants and the acupuncturist who manipulate TEAS will be trained together to ensure identical manipulation. The treatment will be started after randomization.

Participants in this group will receive 15 TEAS sessions in total over a three-week period. TEAS stimulation will be performed using a HANS Acupuncture Point Nerve Stimulator (HANS-100, Huawei Co., Ltd., Beijing, China) through connecting the square-shaped electrode patch (3 × 3 cm) to the acupoint skin. Then, TEAS stimulation will be applied to Ll4 (*He Gu*) and PC6 (*Nei Guan*), ST36 (*Zu San Li*) and SP6 (*San Yin Jiao*) for 30 min each time twice a day for five continuous days followed by a two-day rest interval. The two acupoint groups of the left side will be stimulated in the morning (09:00–11:00 ); those of the right side will be stimulated in the afternoon (14:00–16:00). The TEAS parameters are set as follows: dilatational square wave current output with 2 Hz and 100 Hz alternative frequency (pulse width: 0.6 ms/0.2 ms); intensities in the range of 8–12 mA according to each individual.

#### Sham TEAS group

Participants in the sham TEAS group, which serves as a control group, will also receive 21 days of interval chemotherapy and conventional cancer pain therapy; these therapies are the same as those in the TEAS group. Sham TEAS therapy (lead wires of the apparatus will be cut down) will provided during the trial period. Each step of the sham TEAS manipulation is same as real TEAS without electrical stimulation.

### Outcome measurement

**Fig. 2:**
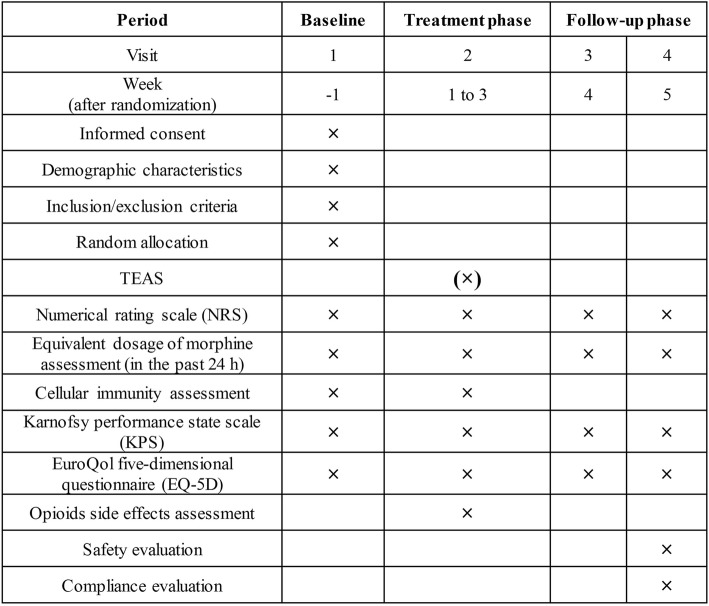
Schedule of treatment and assessment. Outcomes at baseline will be assessed on the day before chemotherapy and TEAS stimulation; outcomes at treatment phase will be assessed on the day after the completion of TEAS or sham TEAS session; outcomes at follow-up phase will be evaluated at the last day of every period. × = all groups; (×) = TEAS groups

The patients will be carefully examined at baseline and reexamined after three weeks of treatment. Follow-up will be performed one and two weeks after completion of the TEAS treatment. Detailed time points of outcome assessments are provided in Fig. 2.

#### Baseline assessments

Baseline assessments will be conducted before randomization, including demographic characteristics (gender, age, height, weight, profession, and education levels of patients, diagnosis, stage of disease, and chemotherapy regimens), pain, equivalent dosage of morphine, cellular immunity function, and life quality.

#### Primary outcome measures

The primary outcome measure in the study is pain assessment demonstrated by Numerical Rating Scale (NRS) scores and equivalent dosage of morphine assessment. The dosage of opioid drugs should be converted to a morphine-equivalent dose according to NCCN Clinical Practice Guidelines in Oncology (Version 2.2011) and the following formulas: transdermal fentanyl (25 mcg/h) ≈ oral oxycodone (30 mg/d) ≈ parenteral morphine (20 mg/day) ≈ oral morphine (60 mg/day).

#### Secondary outcome measures

The secondary outcome measures include: (1) cellular immunity function: immune cell subsets distribution of all participants were detected using a Beckman Coulter FC500 flow cytometer before and after treatment; (2) the Karnofsky Performance Status Scale (KPS) and three-level EuroQol five-dimensional questionnaire (EQ-5D-3 L) for life quality assessment; (3) opioid side effect assessment according to NCCN Clinical Practice Guidelines in Oncology (Version 2.2011); and (4) safety and compliance evaluation.

Any adverse events (AE) and serious AEs occurring during the trial will be recorded. AEs include fainting, severe pain, local infection, unbearable prickling during TEAS treatment, and nervous toxicity due to over-dosage of opioid drugs. All details, such as the date of occurrence, time, degree, and measurement related to the treatment will be documented.

### Quality control

The trial protocol has been modified according to suggestions from experienced acupuncturists and oncologists. Before the trial, all researchers who enroll participants and collect data must attend a series of training sessions. These training sessions will ensure that the research staff involved fully understands the research protocol and standard operating procedures. During the trial, we will establish an inspector to guide and supervise the operators regularly (once every three months). In addition, unified production of various documents and materials, clear and detailed research plans, and assessment indicators and guides will be defined before and during the trial. Economic compensation and free TEAS treatment are also considered as methods for improving compliance. Data management and monitoring will be performed by using ResMan Research Manager (http://www.medresman.org). The principal investigator and the clinical epidemiologist will have access to all the data in the study.

### Sample size calculation

The sample size of this study is estimated using the two proportions comparison method. According to our preliminary test, the average NRS score was decreased 0.051 in the TEAS group and increased 0.585 in the sham TEAS group. The combined standard deviation was 1.260. A single-sided 5% significance level and 90% power were considered; the sample size will be calculated based on the equation below.

$$ n1=n2=\frac{2{\left({u}_a+{u}_{\upbeta}\right)}^2{\upsigma}^2}{\updelta^2} $$

Approximately 67 participants in each group were calculated to be required. Estimating a 20% dropout rate, 160 participants in total will be enrolled with 80 initial participants for each group.

### Statistical analysis

All data in this study will be analyzed by a blinded statistician using the SPSS v20.0 (SPSS, Chicago, IL, USA). Independent sample T test and Chi-square test (χ^2^ test) will be used for numerical variables and categorical variables, respectively. When the distribution of variables is abnormal, a non-parametric test will be selected. A *P* value < 0.05 will be considered statistically significance.

## Discussion

People worldwide are increasingly willing to accept acupuncture treatment given its therapeutic effect on analgesia. As novel types of acupuncture, TEAS or acupuncture-like TENS exert analgesic effects similar to acupuncture [19]. Moreover, TEAS becomes more acceptable than acupuncture given its non-invasive feature [26, 27]. In recent years, research has increasingly focused on the analgesic effect of acupuncture on cancer-related pain. Given the lack of sufficient clinical evidence, whether acupuncture is effective for treating cancer pain in adults remains unclear [4]. Thus, further larger and methodologically sound trials are required [17]. In this trial, the therapeutic advantage of TEAS will also be assessed by investigating the changes on immunosuppression and other side effects result from opioid drugs in cancer pain patients. Cellular immune function will be detected regularly per routine practice in oncology wards, so investigating the immune-regulatory effect of TEAS is feasible.

Although each step of sham TEAS manipulation is the same as that in real TEAS with the exception of electrical stimulation (the lead wires will be cut down), blinding of TEAS treatment seems impossible because patients ultimately know whether they are receiving. To improve participant compliance, all patients enrolled in this trial will receive a 21-day interval of chemotherapy according to NCCN Clinical Practice Guidelines in Oncology and conventional cancer pain therapy under the guidance of the WHO three-step analgesic ladder principle. This process will improve participant compliance. Moreover, free TEAS treatment will further contribute to reducing the dropout rate.

Briefly, the purpose of this trial is to confirm whether TEAS is an effective adjunct to standard cancer pain therapy for moderate and severe cancer-related pain. This study may also confirm the advantage of TEAS on improving immune function, decreasing the dosage of opioid drugs and reducing the occurrence of opioid-related side effects.

## Trial status

Participant enrollment started on 1 November 2014. Enrollment and trial completion are expected to be completed by the end of July 2018.

## Additional file

Additional file 1: SPIRIT 2013 Checklist: Recommended items to address in a clinical trial protocol and related documents*. (PDF 182 kb)

## Abbreviations

EQ-5D: EuroQol five-dimensional questionnaire
KPS: Karnofsy performance state scale
NCCN: National Comprehensive Cancer Network
NRS: Numerical rating scale
RCT: Randomized controlled trial
TEAS: Transcutaneous electrical acupoint stimulation
TENS: Transcutaneous electrical nerve stimulation
UICC: Union for International Cancer Control
